# Resveratrol induces insulin gene expression in mouse pancreatic α-cells

**DOI:** 10.1186/2045-3701-3-47

**Published:** 2013-12-13

**Authors:** Sherwin Xie, Rohit Anthony Sinha, Brijesh K Singh, Guo Dong Li, Weiping Han, Paul M Yen

**Affiliations:** 1 Laboratory of Hormonal Regulation, Program of Cardiovascular and Metabolic Disorders, Duke-NUS Graduate Medical School, 8 College Road, Singapore, Singapore; 2 Department of Clinical Research, Singapore General Hospital, Singapore 169857, Singapore; 3 Laboratory of Metabolic Medicine, Singapore Bioimaging Consortium, A*STAR, Singapore, Singapore

## Abstract

**Background:**

Type 1 and type 2 diabetes are characterized by loss of β-cells; therefore, β-cell regeneration has become one of the primary approaches to diabetes therapy. Resveratrol, a naturally occurring polyphenolic compound, has been shown to improve glycaemic control in diabetic patients, but its action on pancreatic α-cells is not well understood.

**Findings:**

Using mouse α-cells (αTC9), we showed that resveratrol induces expression of pancreatic β-cell genes such as *Pdx1* and *Ins2* in a SirT1-dependent manner. The mRNA and protein levels of insulin were further increased by histone deacetylase (HDAC) inhibition.

**Conclusion:**

In summary, we provide new mechanistic insight into the anti-diabetic action of resveratrol through its ability to express β-cell genes in α-cells.

## Findings

Resveratrol has been shown to improve glycaemic control in humans [1]. Animal studies have shown similar beneficial effects of resveratrol [2] by increasing insulin secretion or enhancing sensitivity to insulin in peripheral organs via activation of SirT1 [3]. Recently, several reports described the ability of pancreatic α-cells to de-differentiate into insulin-producing cells after β-cell loss [4-6]. These findings raise the possibility for new diabetic therapies that exploit α-cell plasticity. In this study, we show that resveratrol can induce expression of several β-cell genes and insulin expression in pancreatic α-cells. Our results shed light on resveratrol action in α-cells and expand our understanding of its anti-diabetic effects.

### Resveratrol induces re-expression of insulin and other pancreatic β-cell genes in a SirT1-dependent manner

**Figure 1:**
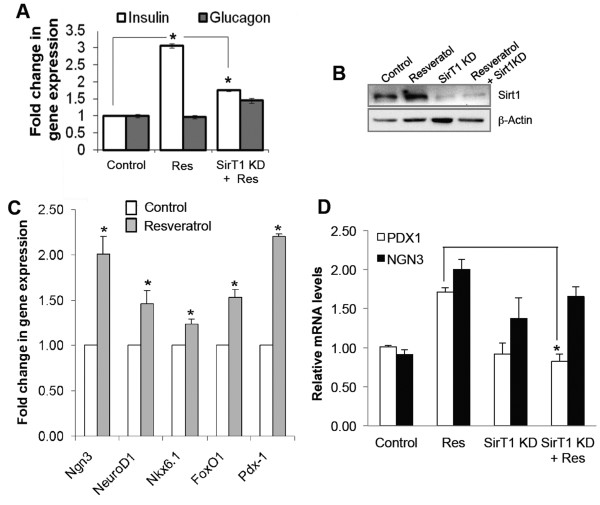
**Resveratrol increase insulin mRNA levels in α-cells via SirT1. (A)** qPCR analysis of *Ins2* and *Gcg* mRNA levels after resveratrol treatment (25 μM / 24 hr) with or without SirT1 KD in α-cells. **(B)** Immunoblot showing efficiency of SirT1 knockdown in α-cells. **(C, D)** qPCR analysis of endocrine progenitor and β-cell specific markers after resveratrol treatment (25 μM / 24 hr). Bars represent the mean of the respective individual ratios ± SEM (n = 3). **(D)** qPCR analysis of *Pdx1* and *Ngn3* mRNA levels after resveratrol treatment (25 μM / 24 hr) with or without SirT1 KD in α-cells. The asterisk indicates P < 0.05.

αTC9 is a subclone selected for high glucagon expression and virtually no insulin expression [7]. Surprisingly, resveratrol significantly increased the expression of mouse *Ins2* mRNA in a SirT1-dependent mechanism in these cells after 24 hr of treatment (Figure 1A, B) while glucagon mRNA was not significantly altered (Figure 1A). Next, we examined the expression of other β-cell markers (*Ngn3*, *NeuroD1*, *Nkx6.1*, *FoxO1* and *Pdx1*) that regulate pancreatic β-cell differentiation and insulin gene transcription in α-cells. Interestingly, resveratrol increased expression of key β-cell transcription factors such as *Pdx1* as well as *Ngn3*, *NeuroD1*, *Nkx6.1* and *FoxO1* (Figure 1C). Similar to its effect on insulin expression, resveratrol’s induction of *Pdx1* was found to be SirT1-dependent whereas *Ngn3* expression did not depend upon SirT1 (Figure 1D).

### Re-expression of insulin gene by resveratrol in α-cells is enhanced by HDAC inhibition

**Figure 2:**
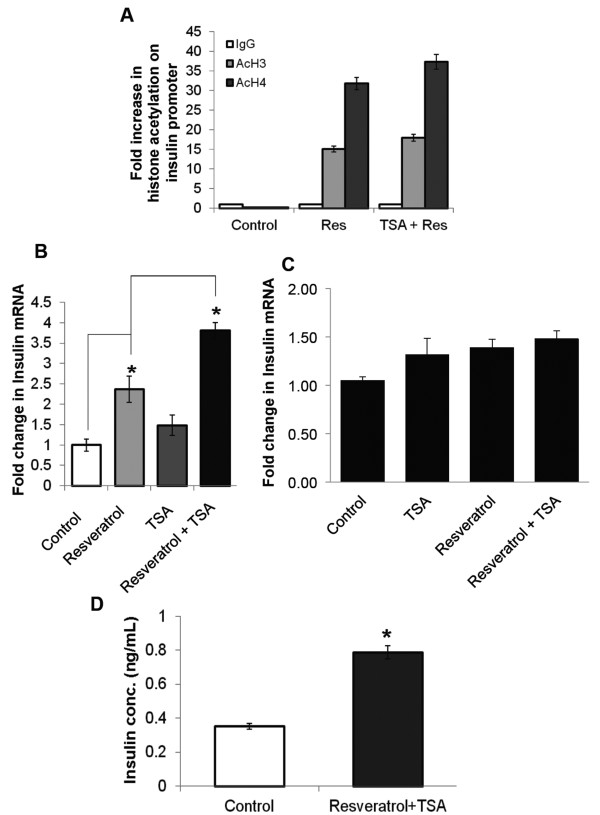
**Resveratrol induced insulin levels are enhanced further by HDAC inhibition. (A)** ChIP-qPCR analysis showing H3/H4 acetylation at *Ins2* promoter after resveratrol alone (25 μM / 24 hr) or TSA (50 nM) co-treatment in αTC9. **(B)** qPCR analysis of *Ins2* mRNA after resveratrol alone (25 μM / 24 hr) or TSA (50 nM / 24 hr) co-treatment in αTC9. **(C)** qPCR analysis of *Ins2* mRNA after resveratrol alone (25 μM / 24 hr) or TSA (50 nM / 24 hr) co-treatment in INS-1 cells. **(D)** RIA measurement of intracellular insulin protein after resveratrol (25 μM / 24 hr) and TSA (50 nM / 24 hr) co-treatment in α-cells. Bars represent the mean of the respective individual ratios ± SEM (n = 3). The asterisk indicates P < 0.05.

Earlier studies of *Pdx1* showed that it induced histone acetylation at the insulin promoter [8]. Therefore we performed ChIP-qPCR for acetylated histone H3 and H4, spanning the enhancer binding site of *Pdx1* in the insulin promoter region. Our results showed a significant increase in H3 and H4 acetylation after resveratrol treatment, which was further enhanced by the co-administration of a HDAC inhibitor, Trichostatin A (TSA) (Figure 2A). This increase in promoter acetylation also correlated with increased transcription of the insulin gene (Figure 2B). We used rat INS-1cells (pancreatic β-cell line) to see the effect of resveratrol and TSA on insulin gene. Interestingly, we observed little or no induction of insulin gene expression by resveratrol and/or TSA in a β-cell line (Figure 2C). This finding suggests that resveratrol and HDAC inhibitors may be more effective in inducing insulin in heterologous cells where it is normally repressed. To validate increased insulin protein expression, RIA was used to quantify the insulin content in α-cells. Although no significant increase in intracellular insulin protein was detectable in resveratrol- or TSA-treated cells (data not shown), there was a significant increase in insulin protein after resveratrol and TSA co-treatment (Figure 2C).

Resveratrol has emerged as a promising anti-diabetic agent that exhibits significant ability to lower serum glucose in diabetic patients [2]. Recent experiments in genetically-manipulated mice have established that α-cells can directly trans-differentiate into β-cells under certain conditions such as β-cell loss in lineage-traced mice [4]. While the induction of β-cell genes such as *Pdx1* can lead to insulin expression in α-cells [8,9], cell transformation leading to expression of β-cell genes is another potential strategy to increase insulin production [5]. In this regard, several new drugs are being developed that modulate α-cell plasticity [10]. Our observation that resveratrol was able to induce insulin synthesis in α-cells is germane since it currently is undergoing clinical trials for treatment of type 2 diabetes.

The insulin-inducing effect on α-cells by resveratrol was SirT1-dependent. Furthermore, the induction of *Pdx1* by resveratrol and the accompanying epigenetic changes on the insulin promoter suggests that it may have a broader reprogramming action than mere stabilization of low abundance insulin mRNA in these cells. In this connection, using an HDAC inhibitor in combination with resveratrol further enhanced insulin induction at both the mRNA and protein levels. In summary, our findings demonstrating the effects of resveratrol on α-cell plasticity provide a new understanding of its anti-diabetic actions and point towards novel treatment strategies for diabetes.

## Materials and methods

### Cell culture

αTC9 cells, a mouse pancreatic α-cell line [7], were grown in DMEM containing 1 g/L glucose, supplemented with 10% FBS, 50 U/mL penicillin and 50 U/mL streptomycin. After adherence, cells were treated with 25 μM resveratrol for 24 hr. SirT1 knockdown was performed using Silencer Select duplex oligo-ribonucleotides targeting mouse SirT1 and a non-targeting control siRNA (Life Technologies, USA). In knockdown studies, resveratrol was added for 24 hr after 2 days of knockdown. Rat INS-1 cells were cultured using standard protocol.

### RNA isolation and real-time PCR (qPCR)

Total RNA was isolated using Invitrap Spin Cell RNA Mini Kit (Stratec Molecular, Germany) and qPCR was performed using the QuantiFast SYBR Green PCR Kit (QIAGEN, USA) according to the manufacturer’s instructions. Samples were normalised to actin. Fold changes were calculated using 2^-ddCt^.

### Western blotting

Cells were lysed using Celytic M mammalian lysis buffer (Sigma-Aldrich, USA) and immunobloting was performed according to manufacturer’s instructions (Bio-Rad, USA). Densitometry analysis was performed using Image J software (NIH, USA).

### Chromatin immunoprecipitation (ChIP)–qPCR analysis

ChIP assays using control rabbit IgG (Santa Cruz, USA), anti-acetylated histone H3 (Abcam, USA) and anti-acetylated histone H4 (Merck-Millipore, USA) were performed using Magna ChIP™ G - Chromatin Immunoprecipitation Kit (Merck-Millipore, USA) according to manufacturer’s instructions. 2 μL of immunoprecipitated DNA or 1% input DNA was used with QuantiFast SYBR Green PCR Kit (QIAGEN, USA) for 40 cycles of qPCR using Rotor-Gene® Q (QIAGEN, USA). Primers used amplify the *Pdx1* binding region (−126 to −296) on the insulin promoter.

### Insulin measurement by radioimmunoassay (RIA)

Cells were lysed and extracted by acid-ethanol and insulin content was assayed by RIA (Linco Research, USA).

### Statistical analysis

Compound treatments were performed in triplicate and repeated at least three times independently using matched controls. The data were pooled and results were expressed as mean ± SEM. The statistical significance of differences (P < 0.05) was assessed by two-tailed student’s t-test.

## Competing interests

The authors declare that they have no competing interests.

## Authors’ contributions

SX, RAS and BKS performed the experiments and analysed the results. RAS and PMY conceived the study. GDL, HW and PMY wrote the manuscript. All authors read and approved the final manuscript.
